# Perception of peer physical examination in two Australian osteopathy programs

**DOI:** 10.1186/s12998-016-0102-2

**Published:** 2016-07-11

**Authors:** Brett Vaughan, Sandra Grace

**Affiliations:** College of Health & Biomedicine, Victoria University, PO Box 14428, Melbourne, VIC 8001 Australia; Institute of Sport, Exercise and Active Living, Victoria University, Melbourne, Australia; School of Health & Human Sciences, Southern Cross University, Lismore, Australia; Education for Practice Institute, Charles Sturt University, Albury, Australia

## Abstract

**Background:**

Peer physical examination (PPE) is an efficient and practical educational approach whereby students can practise their examination skills on each other before commencing clinical practice with actual patients. Little is known about the use of PPE in osteopathy education.

**Methods:**

Students in Year 1 of the osteopathy programs at Victoria University (Melbourne, Australia) and Southern Cross University (Lismore, Australia) completed the Examining Fellow Students and the Peer Physical Examination questionnaires prior to, and at the completion of, their first 12-week teaching session. Descriptive statistics were generated for each questionnaire. The McNemar and sign tests were used to evaluate differences between each questionnaire administration. Logistic regression was used to evaluate the influence of demographics on responses to both questionnaires.

**Results:**

Results showed that students in both programs were generally willing to examine non-sensitive areas both before and after the 12-week teaching session. Students’ were less apprehensive about PPE at the end of the teaching session, and this was reinforced by results for previous exposure to PPE in other courses. Consistent with previous studies, unwillingness to participate in PPE was associated with being female, being born outside Australia, holding religious beliefs, and being older.

**Conclusions:**

This is the first study to explore students’ perceptions of PPE in this cohort and provides a basis for further work, including evaluating longer term changes in student perception of PPE, and whether these perceptions extend to practising manual therapy techniques. This study demonstrates that perceptions about PPE reported in medicine and other disciplines, namely that unwillingness to participate in PPE is associated with being female, being born outside Australia, holding religious beliefs, and being older, also apply to osteopathy. These findings are significant for all manual therapy students who spend a substantial portion of their course developing skills in PPE and practising manual therapy techniques. They highlight the need for curriculum development that acknowledges the importance of good practice in PPE, including discussions about body image, feedback skills training for educators, and providing detailed information to students about what to expect in practical skills classes before they commence their course.

**Electronic supplementary material:**

The online version of this article (doi:10.1186/s12998-016-0102-2) contains supplementary material, which is available to authorized users.

## Background

Peer physical examination (PPE) is widely used in health professional education programs to introduce learners to the physical examination skills required for practice in their chosen profession. PPE is the process where learners practice in pairs or in small groups of fellow learners to develop their skills in the physical examination of patients in preparation for clinical practice.

Much of the PPE literature has focused on medicine with limited literature in other professions including nursing [1], physiotherapy [2] and osteopathy [3]. Numerous benefits for the use of PPE have been described including practising the application of clinical skills prior to patient exposure [4, 5]; developing an appreciation for examining a patient, and being examined [5, 6]; developing professionalism [7]; allowing students to examine a range of body types [6]; receiving peer feedback [8]; and reinforcing anatomy knowledge [5, 9]. Moreover PPE is easy to organise [6] and less expensive than standardised or actual patients. Despite these benefits students can still feel uncomfortable or embarrassed [8]. Their reluctance to participate has been associated with cultural/religious background [8], poor body image [10]; risk of inappropriate body contact [11, 12]; and potential identification of pathologies [6].

Osteopathy is a manual therapy profession that, within the Australian context, focuses on the management of musculoskeletal complaints [13]. During their training, osteopathy students at both institutions participating in the present study learn and practise a range of physical examination skills related to the musculoskeletal system but do not examine intimate body regions [9] beyond the femoral triangle, anterior hip region and chest (excluding breast tissue). They also learn screening examinations for the cardiovascular, respiratory, gastrointestinal and neurological systems, and practise a range of manual therapy techniques. All of these skills are practised in the classroom on fellow students before entering clinical practice with actual patients in the latter years of the program.

Developing clinical skills is an integral part of osteopathy programs [14]. In the programs in the present study, students spend over 300 h learning clinical skills in practical classes over their 5 years of training, typically for between 2–4 h per week. This volume of PPE has yet to be reported in the literature, and may be higher than many non-manual therapy education programs (i.e. medicine, nursing) where PPE has been described. Therefore, understanding students’ perceptions of PPE in manual therapy education programs could be used to inform policies and procedures, not only for practising PPE in class but also for practising manual therapy techniques on their peers, an activity that has received little attention in the literature to date.

It may be possible to extrapolate the findings from PPE studies in medicine to osteopathy, however as Wearn et al. [1] suggest students may ‘… begin their programme with a slightly different world view’ (p. 885). Consorti et al. [3], in their study comparing PPE perceptions of Italian medical and osteopathy students, posited that the latter are likely to enter their program of study with a preconceived idea about body contact and *learning to touch* as a part of their training. These authors compared the perceptions of PPE in Italian medical and osteopathy students, demonstrating that the latter students were more positive about their PPE experience, particularly the part-time students. Osteopathy students in the Consorti et al. [3] study were either full-time or part time, with the part time students already having completed training as a health professional (typically medicine or physiotherapy) prior to entering the osteopathy program. In contrast, Australian osteopathy students complete their training in a full-time program, albeit they may enter with a previous health profession qualification. This difference, in part, limits the comparisons that can be drawn between the Australian and Italian training context.

PPE and practising manual therapy techniques on peers is a traditionally accepted part of training to be an osteopath, however there is very little literature that investigates student perceptions of these practices. The aim of the present study was to explore the perceptions of osteopathy students in two Australian teaching programs before and after their exposure to PPE activities over a 12-week teaching period to ascertain whether these perceptions are consistent with the literature on PPE in other health professions, and to inform curriculum development in osteopathy and other health professions.

## Methods

The study was approved by the Victoria University and Southern Cross University Human Research Ethics Committees (ECN15-007).

### Participants

Year 1 students enrolled in the osteopathy programs at Victoria University (VU) and Southern Cross University (SCU) received an email inviting them to participate in the study. Participation was voluntary and responses were anonymous. Students self-generated a code that would allow the matching of pre- and post-responses.

The curriculum at VU in the first 12-week teaching encompasses PPE activities that relate to the musculoskeletal examination, surface anatomy and manual therapy techniques for the upper extremity, cervical spine, and head and face. The thoracic and lumbar spine, and the lower extremity are covered in the second 12-week teaching period. At SCU students cover PPE activities that relate to the musculoskeletal examination, surface anatomy and manual therapy techniques for the upper and lower extremity during the first 12-week teaching period. At both institutions verbal consent to participate is required during each session, as recommended by Wearn and Bhoopatkar [15].

### Measures

Participants were asked to complete a demographic questionnaire, the *Examining Fellow Students* (EFS) questionnaire [11] and the *Peer Physical Examination* questionnaire (PPEQ) [3]. The demographic questionnaire asked participants to indicate their age, biological gender, whether they were born in Australia, previous participation in a course involving physical examination, whether they currently practised a religion, and whether English was the primary language spoken at home. These demographic characteristics are consistent with those explored in the previous PPE literature [16].

### Intervention

Reid et al. [17] and Hendry [16] suggest evaluating student perceptions of PPE before and after their first PPE experience. Consequently, participants were asked to complete the questionnaires before their first practical skills class at the start of the 2015 academic year (February) (T1) and again at the end of the first 12 week teaching session (T2). Participants were only required to complete the demographic questionnaire at T1.

### Data analysis

Descriptive statistics were generated for each of the demographics, EFS and PPEQ. The McNemar test was used to examine differences between the EFS categorical responses at week 1 and week 12. This test is used to evaluate paired nominal data. As the PPEQ data were ordinal and not assumed to be interval-level data [18], the sign test was used to examine the differences between administrations for each item at T1 and T2. Alpha for both questionnaires was set at *p* < 0.05. Effect sizes for the PPEQ were also calculated using the formula *r* = Z/√N, where Z is the Z score and N is the total sample size [19]. The effect sizes were interpreted as small (0.1), medium (0.3) and large (0.5) [19]. While interpreted in a similar way, the effect size calculation in the present study is not Cohen’s *d*.

The relationship between the demographics and the responses to the EFS and PPEQ were examined with binomial logistic regression and ordinal logistic regression respectively, using the *rms* package (version 4.4-0) [20] in *R* (version 3.2.2) [21]. Backwards elimination with the Akaike criterion (AIC) as the cutoff was used to identify significant variables in each model. Odds ratios (OR) were calculated for significant variables and interpreted according to Hopkins [22]. Internal consistency of the PPEQ was calculated at T1 and T2 with both Cronbach’s alpha and McDonald’s omega [23, 24] using a polychoric correlation in the *R* program [21] with the *psych* package (version 1.5.8) [25].

## Results

Responses rates at T1 were 86 % (*n* = 114) and 91 % (*n* = 41) from VU and SCU respectively. At T2, response rates were 76 % (*n* = 101) and 67 % (*n* = 29). Matched T1 and T2 data were available for 105 students, and it is this data set that is analysed here. Matched data from VU made up 81.9 % (*n* = 86) of that analysed in the present study. Demographic data are presented in Table 1.

**Table 1:** Demographics by institution

	VU	SCU
Age		
Mean (SD)	20 years (±3.17)	27.5 years (±9.73)
Range	18–33 years	18–52 years
Biological gender		
Male	46 (53.5 %)	7 (36.8 %)
Female	39 (45.3 %)	12 (63.2 %)
Previous course involving PPE		
Yes	12 (14 %)	7 (36.8 %)
No	73 (84.9 %)	12 (63.2 %)
Born in Australia		
Yes	82 (95.3 %)	15 (78.9 %)
No	3 (3.5 %)	4 (21.1 %)
English as primary language at home		
Yes	84 (97.7 %)	18 (94.7 %)
No	1 (1.2 %)	1 (5.3 %)
Practice a religion		
Yes	17 (19.8 %)	1 (5.6 %)
No	68 (79.1 %)	17 (89.5 %)

### Examining Fellow Students (EFS) questionnaire

EFS responses for all 105 students were not significantly different between T1 and T2 for both willingness to examine all body regions on a peer, or to be examined by a peer.

#### Demographics and the EFS

At T1, all SCU students were willing to examine all of the listed areas on a fellow student regardless of biological gender (Table 2). In contrast, a small number of students from VU indicated that were unwilling to examine numerous areas on the opposite biological gender, and in some cases, both biological genders. With regard to being examined by a peer, VU students reported being unwilling to be examined in the groin area by a peer of the opposite biological gender (*n* = 8, 9.3 %). All SCU students indicated a willingness to have all regions examined by their peers regardless of biological gender. None of the demographics were significant in the regression models for both being examined by, or examining, a peer.

**Table 2:** Examining Fellow Students questionnaire responses at T1 by institution

	VU				SCU			
Examine a peer	Willing	Same gender	Different gender	Both same and different gender	Willing	Same gender	Different gender	Both same and different gender
Head and neck	86 (100 %)				100 %			
Hands	84 (97.7 %)		2 (2.3 %)		100 %			
Arm and shoulder	85 (98.8 %)		1 (1.2 %)		100 %			
Upper body (no breast exposure)	85 (98.8 %)		1 (1.2 %)		100 %			
Abdomen	85 (98.8 %)		1 (1.2 %)		100 %			
Back	85 (98.8 %)		1 (1.2 %)		100 %			
Groin (without genital exposure)	78 (91.0 %)		4 (4.5 %)	4 (4.5 %)	100 %			
Feet	84 (97.7 %)		1 (1.2 %)	1 (1.2 %)	100 %			
Legs	85 (98.8 %)		1 (1.2 %)		100 %			
Hips	85 (98.8 %)		1 (1.2 %)		100 %			
Chest (no breast exposure)	84 (97.7 %)		2 (2.3 %)		100 %			
Pelvis (without genital exposure)	83 (96.5 %)		1 (1.2 %)	2 (2.3 %)	100 %			
Be examined by a peer	Willing	Same gender	Different gender	Both same and different gender	Willing	Same gender	Different gender	Both same and different gender
Head and neck	85 (98.8 %)		1 (1.2 %)		100 %			
Hands	85 (98.8 %)		1 (1.2 %)		100 %			
Arm and shoulder	85 (98.8 %)		1 (1.2 %)		100 %			
Upper body (no breast exposure)	84 (97.7 %)		1 (1.2 %)	1 (1.2 %)	100 %			
Abdomen	83 (96.5 %)		1 (1.2 %)	2 (2.3 %)	100 %			
Back	85 (98.8 %)		1 (1.2 %)		100 %			
Groin (without genital exposure)	76 (87.2 %)		8 (8.1 %)	2 (2.3 %)	100 %			
Feet	84 (97.7 %)		1 (1.2 %)	1 (1.2 %)	100 %			
Legs	85 (98.8 %)		1 (1.2 %)		100 %			
Hips	85 (98.8 %)		1 (1.2 %)		100 %			
Chest (no breast exposure)	83 (96.5 %)		2 (2.3 %)	1 (1.2 %)	100 %			
Pelvis (without genital exposure)	83 (96.5 %)		1 (1.2 %)	1 (2.3 %)	100 %			

At T2, two SCU students indicated they were unwilling to have their chest examined by an opposite biological gender peer, and examine the pelvis of peers of either biological gender (Table 3). None of the demographic variables were significant in the regression model.

**Table 3:** Examining Fellow Students questionnaire responses at T2 by institution

	VU				SCU			
Examine a peer	Willing	Same gender	Different gender	Both same and different gender	Willing	Same gender	Different gender	Both same and different gender
Head and neck	86 (100 %)				100 %			
Hands	84 (97.7 %)		2 (2.3 %)		100 %			
Arm and shoulder	85 (98.8 %)		1 (1.2 %)		100 %			
Upper body (no breast exposure)	85 (98.8 %)		1 (1.2 %)		100 %			
Abdomen	85 (98.8 %)		1 (1.2 %)		100 %			
Back	85 (98.8 %)		1 (1.2 %)		100 %			
Groin (without genital exposure)	78 (91.0 %)		4 (4.5 %)	4 (4.5 %)	100 %			
Feet	84 (97.7 %)		1 (1.2 %)	1 (1.2 %)	100 %			
Legs	85 (98.8 %)		1 (1.2 %)		100 %			
Hips	85 (98.8 %)		1 (1.2 %)		100 %			
Chest (no breast exposure)	84 (97.7 %)		2 (2.3 %)		100 %			
Pelvis (without genital exposure)	83 (96.5 %)		1 (1.2 %)	2 (2.3 %)	100 %			
Be examined by a peer	Willing	Same gender	Different gender	Both same and different gender	Willing	Same gender	Different gender	Both same and different gender
Head and neck	86 (100 %)				19 (100 %)			
Hands	86 (100 %)				19 (100 %)			
Arm and shoulder	86 (100 %)				19 (100 %)			
Upper body (no breast exposure)	86 (100 %)				19 (100 %)			
Abdomen	85 (98.8 %)		1 (1.2 %)		19 (100 %)			
Back	86 (100 %)				19 (100 %)			
Groin (without genital exposure)	84 (97.7 %)			2 (2.3 %)	19 (100 %)			
Feet	85 (98.8 %)			1 (1.2 %)	19 (100 %)			
Legs	86 (100 %)				19 (100 %)			
Hips	86 (100 %)				19 (100 %)			
Chest (no breast exposure)	85 (98.8 %)			1 (1.2 %)	18 (94.7 %)		1 (5.3 %)	
Pelvis (without genital exposure)	85 (98.8 %)			1 (1.2 %)	18 (94.7 %)			1 (5.3 %)

#### Peer Physical Examination Questionnaire (PPEQ)

Descriptive and inferential statistics for the PPEQ items are presented in Additional file 1. Median values for PPEQ items 1 through to 12 were all significantly different from T1 to T2 (*p* < 0.02). Median values for these items either improved from T1 to T2 or were stable across the two administrations of the questionnaire, and effect sizes (r) ranged from 0.24 to 0.55. Items 13 to 16 were not significantly different from T1 to T2 (*p* > 0.05).

##### Demographics and the PPEQ items

Results of the ordinal regression models for the PPEQ items at T1 and T2 are in Additional file 2. Speaking English at home was not included in the modelling as only one participant was in this category. Practising a religion, being born outside Australia, biological gender, age, and having studied a previous course involving PPE were related to a number of the PPEQ items. There were no significant associations between the demographic variables and item 1 *In general, I (will) feel comfortable when performing PPE on a colleague of mine*, item 11 *I (will) feel comfortable when PPE is performed on me by a colleague of the opposite sex than mine*, item 13 *To perform PPE is an appropriate practice for the education of an osteopath*, item 14 *To undergo PPE is an appropriate practice for the education of an osteopath,* and item 16 *It is a sign of professionalism as a student to accept to perform and undergo PPE*, at either T1 and T2.

##### Religion

Practising a religion was only significant for item 5 *I am concerned of being a possible object of sexual interest during PPE* (OR 4.95, *moderate*), item 6 *I am concerned of experiencing possible sexual interest for my colleagues during PPE* (OR 3.28, *small*), and item 12 *It is inappropriate to perform PPE on persons that will be my future colleagues* (OR 3.18, *small*) at T1. Those students practising a religion were less likely to agree with these items. Practising a religion was not significant for any PPEQ item at T2.

##### Born outside of Australia

Being born outside Australia was significant at both T1 and T2 for item 3 *I (will) feel embarrassed if I am undressed for PPE in front of my group of colleagues* (OR 2.98, *small* & OR 13.19, *large*), item 5 *I am concerned of being a possible object of sexual interest during PPE* (OR 4.95 & OR 4.64, *moderate*), and item 7 *I am concerned of experiencing possible sexual interest for my teacher or tutor during PPE* (OR 7.24, *moderate* & OR 9.11, *large*). At T2, being born overseas was significant for item 4 *I will feel embarrassed if I am undressed for PPE in front of my teacher or tutor* (OR 9.20, *large*). However, all of these ORs exhibited large 95 % confidence intervals.

##### Biological gender

Female students were more likely to agree with item 5 *I am concerned of being a possible object of sexual interest during PPE* (OR 2.66, *small*) at T1, however this was not significant at T2. At T1 and T2, item 3 *I (will) feel embarrassed if I am undressed for PPE in front of my group of colleagues* (OR 2.91, *small* & OR 1.91, *small*), item 4 *I will feel embarrassed if I am undressed for PPE in front of my teacher or tutor* (OR 2.88, *small* & OR 1.84, *small*), and item 15 *In performing PPE I will get useful feedback from my colleagues about my skills* (OR 2.07, *small* & OR 1.93, *small*) were all significant for female students. These students were more likely to agree with items 3 and 4, and more likely to disagree with item 15.

##### Age

For the regression analysis, student age was categorised with age 18–19 years as the comparator. At T1, item 6 *I am concerned of experiencing possible sexual interest for my colleagues during PPE* was significant for the 20–25 year age group (OR 1.95, *small*), and 26 years and over group (OR 4.48, *moderate*) with both groups being more likely to agree with this statement. There was no association between age and this item at T2. At T2, age was significantly associated with items 8 to 11 with OR’s ranging from 1.06 to 6.23 (Additional file 2). These older age groups were less likely to agree with these items than those in the 18 to 19 year age group.

Multiple items were significant at both T1 and T2. For item 5 *I am concerned of being a possible object of sexual interest during PPE* (OR 1.39 & OR 2.71, *small*), those students aged between 20 and 25 years were more likely agree with this statement at both time points. In contrast, those 26 years or over were more likely to agree with this item at T1 (OR 21.11, *large*), but less likely at T2 (3.63, *moderate*). For item 7, *I am concerned of experiencing possible sexual interest for my teacher or tutor during PPE,* students aged over 20 years were more likely to agree with this item at T1 and T2 (Additional file 2). Conversely for item 12, students aged over 20 years were less likely to agree with item 12 *It is inappropriate to perform PPE on persons that will be my future colleagues* (Additional file 2), that is, they see that PPE is appropriate to perform on future colleagues.

##### Previous course involving PPE

Only item 3 *I (will) feel embarrassed if I am undressed for PPE in front of my group of colleagues* was significant at T1 (OR 2.07, *small*), and not significant at T2. Students who had undertaken a previous course that involved PPE were less likely to agree with this item.

#### Internal consistency

Internal consistency of the PPEQ was acceptable at T1 (Cronbach’s alpha = 0.92, McDonald’s omega = 0.70). At T2 Cronbach’s alpha was acceptable (0.92). However, McDonald’s omega was slightly below an acceptable level (0.69). The Cronbach’s alpha scores for the PPEQ at T1 and T2 did not improve if an item was removed during the calculation.

## Discussion

The aim of the present study was to explore perceptions of first year osteopathy students at two Australian universities about PPE. Overall, students in the present study were willing to examine, and have examined all body regions listed in the questionnaire. This is consistent with results of another study [12] and within the 5 % range of students unwilling to participate in PPE identified by Power and Center [26]. The only region where this value was larger was for students from VU who indicated an unwillingness to examine the groin of a peer, or have their groin examined, in some cases, regardless of peer biological gender. Students in the present study were less apprehensive about PPE and perceived it as a professional experience, as did those in the study reported by Consorti et al. [3]. The findings of the present study, for the first time, reinforce the anecdotal experiences of the authors with the application of PPE in osteopathy.

The concept of PPE in osteopathy education extends beyond the rehearsal and development of physical examination skills to include the application of osteopathic manipulative therapy (OMT) and other manual therapy techniques. The World Health Organisation [14] *Benchmarks for Training in Osteopathy* requires programs to graduate students with:

- *competency in the palpatory and clinical skills necessary to diagnose dysfunction in the aforementioned systems and tissues of the body, with an emphasis on osteopathic diagnosis;*
- *competency in a broad range of skills of OMT;*
- *proficiency in physical examination and the interpretation of relevant tests and data, including diagnostic imaging and laboratory results.*

Achieving these benchmarks requires substantial time practising these skills on peers in the classroom. In the context of the present study, over the 12-week teaching period, students spent approximately 50 h in a PPE environment. By the completion of their program of study they will have spent approximately 300 h developing their practical skills in the classroom and 1000 h with actual patients in a student teaching clinic. It is anticipated, as Rees et al. [27] suggested, that the positive perceptions of PPE identified in this study will remain throughout the entire program. Exposure to the living body early in a students’ training is likely to have a significant influence on the relative ease that students will have with ‘therapeutically touching’ a patient in their clinical training years, and contribute to the development of professional attitudes towards patients [28]. The practice of osteopathy in Australia is focused on the management of musculoskeletal complaints [13]. Therefore there is little need to learn, or be able to practise, examination of sensitive areas like the breast and genitals [29] which are beyond the scope of practice of osteopaths in Australia. Consequently, these regions were not included in the EFS questionnaire.

To be able to develop the manual therapy skills to become a registered osteopath in Australia, students undertake carefully scaffolded and supervised practice throughout their course. Students require full information about what is expected of them before they enrol and explanations about the benefits of participating in PPE need to be made clear [4, 30]. However, some students may not wish to participate in a particular PPE activity, or may place conditions on their participation [5]. Alternative learning pathways such as practising on standardised patients or on family members need to be made available [16]. One of the challenges for educators is to design activities that meet the required learning outcomes while respecting the right of students to refrain from participating. In the history of both osteopathy teaching programs, few challenged have been reported, most being resolved by allowing students to practise initially with someone with whom they feel comfortable (e.g. a student of their own choosing, or a family member or friend), before practising with other students who can provide a wide variety of body types, and personal and medical histories.

No national or international guidelines could be located to guide good practice in PPE and practising treatment techniques on peers. The University of Queensland Medical School [31] called for development of such guidelines in medicine. In osteopathy and other health sciences, guidelines for good practice are likely to include:

- obtaining informed consent from students before they participate in PPE [4, 30];
- telling students what to expect in practical classes before they commence their courses [6, 30, 32];
- facilitating discussions about ethical, cultural and professional issues associated with PPE (e.g. therapeutic touch vs sexual touch; body image; age, gender, cultural influences on willingness to participate in PPE);
- allowing students to choose who they practise with; and
- offering alternatives to students who choose not to participate [16].

### Demographic influences on PPE

The EFS questionnaire asks students to indicate which areas of the body they would not be willing to examine on a peer or have examined by a peer. Numerous authors have reported that students are more willing to examine, rather than be examined by, a peer [11, 33, 34], and this appeared to be consistent with the present study. Students entering an osteopathy program are likely to be aware that their course will include a substantial amount of time devoted to learning clinical assessments and manual therapy skills [3]. Such an assertion is supported by the PPEQ responses in the present study where students were likely to *agree* or *strongly agree* with the items at T1, with either an increase in the median value, or at least with the value remaining high, at T2. Medium to large effect sizes were noted for PPEQ items 1 to 12. The changes in items 1 to 12 from T1 to T2 may reflect ‘reasoned or rationalized changes’ [35] in the students’ perceptions following participation in PPE activities.

The last four PPEQ items relate to the application of PPE in osteopathy education. No significant difference between T1 and T2 was observed for these items (items 13 to 16). Students from both institutions potentially saw PPE as an integral part of their osteopathy education before entering the course, similar to the medical students reported by Chang and Power [12]. Previous studies have found that students’ negative perceptions of PPE may be related to experiences with tutors and classmates. It is hypothesised that the tutors and lecturers of students in the present study may have created a supportive learning environment that contributed to the increase in median values for these items. Such supportive environments incorporate appropriate feedback from lecturers/tutors and peers. Chang and Power [12] found that receiving feedback from peers was a key positive feature of PPE. In the present study, females were less likely to agree with item 15 *In performing PPE I (will) get useful feedback from my colleagues about my skill* at both T1 and T2, however, these OR’s were small.

#### Biological gender

Consistent with findings from other authors is the greater willingness to examine, or be examined by, a peer of the same biological gender [33]. In the present study, there were no significant differences between the pre- and post-test EFS responses. Work by Rees [32] suggested that female students ‘… may also be more likely than males to fear critical and teasing comments…’ (p. 801) and this could account for the less positive perception of feedback from peers in PPE activities. Although both teaching programs aim to incorporate peer feedback as part of the classroom environment where PPE is employed, it may be that further work is required to reinforce this, along with specific training for lecturers and tutors on feedback skills.

Females were also more likely to feel uncomfortable with getting undressed for PPE activities at T1 and T2 although this influence of biological gender was reduced at T2. This result is consistent with the discussion by Rees [32] who used a feminist theory lens to highlight the potential for body image issues to play a role in PPE. Of note is that females were still significantly more likely to feel embarrassed if disrobed in their practical skills class at T2, even though they had experienced 12 weeks of the learning environment and could arguably be more comfortable participating in it. This result highlights the ongoing need to consider body image wherever PPE is employed, including incorporating information about body image in the curriculum before and during the use of PPE, as well as reinforcing the need to demonstrate appropriate draping [6, 36]. Discussions about body image could form part of students’ introduction to PPE.

#### Age

Rees et al. [5] previously demonstrated that older students are less comfortable with PPE. In the present study age was not associated with an unwillingness to examine, or be examined by, a peer [12, 33, 34]. Results from the EFS for age were not significant in the binomial logistic regression models for willingness to examine, or have examined, specific body regions. However, with regard to the PPEQ items, the responses from students aged over 20 were in many cases likely to differ from their peers aged 18–19 years. Most of the OR’s for the PPEQ items were small to moderate, suggesting age is likely to influence a students’ perception of PPE, albeit minimally. Items 8, 9 and 10 were significant for age at T2 but not at T1, and all of these items were those that evaluated whether the student felt comfortable with PPE (Additional file 2). Older students were less likely to agree with these items at T2 suggesting their perception may have become more negative after participating in PPE activities. The only exception was item 11 *I (will) feel comfortable when PPE is performed on me by a colleague of the opposite sex than mine*. Age was not significant for this item at T1 or T2 suggesting that age is unlikely to influence perception of PPE if performed by a colleague of the opposite biological gender.

The apparently conflicting results from our two questionnaires could be related to sample size: only a small number of people reported feeling uncomfortable examining, or having examined, most regions, most commonly the pelvis. Alternatively, the results may simply reflect the different purposes of the two questionnaires: the EFS targets students’ perceptions about PPE of specific body regions. The PPEQ on the other hand draws out responses to the wider learning environment for PPE and students’ level of comfort in it. This global willingness to engage in PPE is not elicited from the EFS.

#### Religion

In the EFS, practising a religion was not significant in the binomial logistic regression models for willingness to examine, or have examined, specific body regions. From the PPEQ data, students who reported practising a religion were initially concerned about being of ‘sexual interest’ to their peers and tutors, however this influence was not present at T2. Those students practising a religion were also more likely to agree with item 12 related to inappropriateness of performing PPE on future colleagues. Again, this influence was not present at T2. These initial perceptions may be influenced by their limited knowledge of PPE at T1. Further, the classes where PPE is employed may be conducted in a professional, sensitive manner, and the students will have experienced this by the time they completed the questionnaires at T2. PPE activities are also taught by both male and female tutors at the two institutions in the present study, and this may reduce the influence of tutor biological gender on student concerns about PPE [32].

A previous study [5] suggested that practising a religion influenced willingness to examine the groin or feet of a peer - this was not the case in the present study, according to EFS data. The current data did not include details of particular religions practised by student(s) and did not explore whether specific religious beliefs accounted for the results [27]. Further, it would be interesting to explore students’ understanding at T1 of what each of the EFS body regions meant to them given that a small number of students indicated this was a body region that they felt uncomfortable examining, having examined, or both. For example, examination of the groin and anterior hip are closely related in a musculoskeletal examination, and the use of the term ‘groin’ may have a particular meaning for an individual, albeit the EFS explicitly states ‘no genital exposure’. The femoral triangle, anterior hip, and chest (excluding breast tissue) are the only potentially sensitive regions of the body that are examined by students in Australian osteopathy programs.

#### Previous course involving PPE

Having previously undertaken a course that involved PPE influenced responses to item 3 *I will feel embarrassed if I am undressed for PPE in front of my group of colleagues* at T1, and item 5 *I am concerned of being a possible object of sexual interest during PPE* at T2. In both instances students were less likely to agree with these two items. This suggests that students who have had previous exposure to PPE are less likely to feel embarrassed, and highlights the importance of providing sufficient information to students before they start their course so that they know exactly what to expect in practical classes, a sentiment commonly called for in the PPE literature [6, 30, 32].

#### Born outside Australia

Being born in Australia influenced responses to a number of PPEQ items, albeit the 95 % confidence intervals for these OR’s were large. Item 3 *I will feel embarrassed if I am undressed for PPE in front of my group of colleagues,* item 5 *I am concerned of being a possible object of sexual interest during PPE,* and item 7 *I am concerned of experiencing possible sexual interest for my teacher or tutor during PPE* were influenced by whether a student was born overseas at both T1 and T2. Those students who were not born in Australia were more likely to agree with these items. It is not possible to isolate these responses to particular countries from the data collected. Therefore feeling embarrassed about being disrobed and possible perception of sexual interest may relate to social influence (non-Anglo Saxon background) [27, 30].

#### Statistical considerations

The internal consistency of the PPEQ was evaluated using two approaches: Cronbach’s alpha and McDonald’s omega [23]. Authors have argued that Cronbach’s alpha may not provide an accurate indication of internal consistency, and McDonald’s omega may be a better option [24, 37, 38]. The PPEQ internal consistency was very good when using alpha, but borderline when using omega. Given the questionnaire has only been used in one other study [3], further work to investigate the psychometric properties of the PPEQ is required. McLachlan et al. [35] have asked authors investigating longitudinal changes in PPE perceptions to provide support for the pre-post differences obtained. In the present study this is provided by the reporting of effect sizes, something that the majority of PPE studies have not done. Many of the effect sizes in the present study are interpreted as medium [19], suggesting that there is likely to be a change pre to post participation in PPE activities but larger participant numbers are required to confirm the results.

#### Limitations

The limitations of this study include its limited longitudinal nature (only a 12 week teaching period) and the fact that not all body regions had been examined by the students before the conclusion of the study period. Further, the study was conducted in two Australian teaching programs with a single cohort, therefore the generalisability to other non-United States osteopathy programs, and other Australian osteopathy student cohorts is limited. Matched data were only available for 105 students. It is possible that some students were not able to complete the questionnaire at either T1 or T2 due to an absence, or had withdrawn from the teaching program prior to completing the questionnaires at T2. The ratio of respondents was approximately 4.5:1 for VU and SCU and this may have influenced some of the results, however it would be difficult to control given the substantial differences in cohort sizes at the two institutions.

Only quantitative data were collected in the present study, and the addition of a qualitative component may shed further light on some of the issues faced by osteopathy students when entering a program of study that emphasises PPE. In particular, previous studies have highlighted the issue of harming, or being harmed by a peer during PPE [5, 7, 33], something which is not captured in either questionnaire employed in the present study. The application of manual therapy techniques carries a very small risk of an adverse reaction [39], so fear of harm may be a valid concern. How the results of the present study relate to the practice of OMT and other treatment techniques would require further investigation. This is particularly relevant as students in osteopathic programs in Australia will be required to practise manual techniques on some body areas that students in the present study were either unwilling to examine, or have examined on themselves. This is an avenue for further work in osteopathy and other manual therapy professions.

Direct comparisons with the Consorti et al. [3] study at item level are not possible as detailed data from the previous study were not available. Such comparisons in future studies will enable a deeper understanding of the quantitative data derived from the PPEQ. The results of the present study also highlight a potential issue with the PPEQ in that it may be subject to a ceiling effect and not necessarily sensitive enough to detect a change in student perceptions. This may have been reinforced in the present study as the analysis was undertaken using the ordinal data generated by the responses to the PPEQ, rather than making the assumption that the underlying data were interval in nature, and subsequently using parametric inferential statistics [18]. The use of these statistics may have yielded different results, however it may have also provided a less accurate indication about pre-post differences. Another factor that influences the interpretation of the PPEQ data is the large 95 % confidence intervals for some of the demographic variables (Additional file 2). In some cases these were quite substantial and suggest that further work is required to confirm if these demographic variables do in fact have a significant influence on the PPEQ responses.

Only one student reported not speaking English at home in the present study. Therefore it is not possible to describe its influence on perception of PPE. It is also possible that other unmeasured factors influence a students’ perception of PPE. The learning environment, interpersonal experiences with the class tutors and peers, learning approach, personality, body image, and motivations for learning could all influence students’ perceptions of PPE. These require exploration in future research. Rees [32] also suggested that tutor biological gender is an avenue for further research and will be considered in future studies.

## Conclusions

PPE is used extensively in Australian osteopathy teaching programs. This is the first study to explore students’ perceptions of PPE in this cohort. Australian osteopathy students are generally willing to participate in PPE. Students saw PPE as an important and relevant part of their training both before and after participation in classroom activities involving PPE. Students who had previously studied a course involving PPE were slightly more positive about PPE than those who had not. Willingness to participate in PPE was associated with biological gender: females were more likely to feel embarrassed when disrobed in practical classes. Being born outside Australia, and holding religious beliefs were also associated with reluctance to participate in PPE. Students over 20 years of age were initially more concerned about being of sexual interest and about performing PPE on a colleague than 18–19 year olds, and generally less likely to feel comfortable about performing PPE after exposure to the course. Further work is required to validate the results of the present study and ultimately to develop evidence-informed, safe, ethical and culturally-sensitive approaches to PPE in Australian osteopathy programs.

The present study adds to the PPE literature by evaluating the perceptions of osteopathy students who spend a substantial portion of their education practising PPE, evaluations that are likely to be common to all manual therapy students. Further, the study highlights a number of important considerations for curriculum development, such as incorporating discussions about body image, feedback skills training for educators, and providing detailed information to students about PPE before they commence their studies.

## Additional files

Additional file 1 Descriptive & inferential statistics for the Peer Physical Examination Questionnaire (PPEQ). (DOCX 100 kb) Additional file 2 Ordinal Logistic Regression for Peer Physical Examination Questionnaire (PPEQ) items & demographics (DOCX 24 kb)
