# Supplementary material for: Perception of peer physical examination in two Australian osteopathy programs
Source: Chiropr Man Therap. 2016 Jul 11;24:21. doi: 10.1186/s12998-016-0102-2 (PMC4939704; doi:10.1186/s12998-016-0102-2)
Supplement: Additional file 1 — Descriptive & inferential statistics for the Peer Physical Examination Questionnaire (PPEQ). (DOCX 100 kb) [file 12998_2016_102_MOESM1_ESM.docx]

**Descriptive & inferential statistics for the Peer Physical Examination Questionnaire (PPEQ).**

|  | **T1** | | **T2** | |  |  |
| --- | --- | --- | --- | --- | --- | --- |
|  | **Mean (SD)** | **Median (Range)** | **Mean (SD)** | **Median (Range)** | **p-value for Sign test** | **Effect size (r)** |
| 1. In general, I (will) feel comfortable when performing PPE on a colleague of mine | 3.31 (0.67) | 3 (2-4) | 3.70 (0.49) | 4 (3-4) | <0.001 | 0.49 |
| 2. In general, I (will) feel comfortable when a colleague performs PPE on me | 3.24 (0.67) | 3 (1-4) | 3.69 (0.61) | 4 (0-4) | <0.001 | 0.56 |
| 3. I (will) feel embarrassed if I am undressed for PPE in front of my group of colleagues | 2.57 (1.24) | 3 (0-4) | 2.98 (1.03) | 3 (0-4) | <0.001 | 0.43 |
| 4. I (will) feel embarrassed if I am undressed for PPE in front of my teacher or tutor | 2.64 (1.22) | 3 (0-4) | 3.04 (1.03) | 3 (0-4) | <0.001 | 0.37 |
| 5. I am concerned of being a possible object of sexual interest during PPE | 3.39 (0.83) | 4 (0-4) | 3.64 (0.68) | 4 (1-4) | 0.002 | 0.29 |
| 6. I am concerned of experiencing possible sexual interest for my colleagues during PPE | 3.35 (0.82) | 4 (1-4) | 3.96 (0.66) | 4 (1-4) | <0.001 | 0.38 |
| 7. I am concerned of experiencing possible sexual interest for my teacher or tutor during PPE | 3.68 (0.61) | 4 (1-4) | 3.84 (0.43) | 4 (2-4) | 0.014 | 0,24 |
| 8. I (will) feel comfortable when performing PPE on a colleague of my same sex | 3.57 (0.60) | 4 (2-4) | 3.81 (0.54) | 4 (0-4) | <0.001 | 0.34 |
| 9. I (will) feel comfortable when performing PPE on a colleague of the opposite sex than mine | 3.29 (0.74) | 3 (1-4) | 3.68 (0.53) | 4 (2-4) | <0.001 | 0.45 |
| 10. I (will) feel comfortable when PPE is performed on me by a colleague of my same sex | 3.55 (0.60) | 4 (2-4) | 3.81 (4.18) | 4 (2-4) | <0.001 | 0.36 |
| 11. I (will) feel comfortable when PPE is performed on me by a colleague of the opposite sex than mine | 3.3 (0.75) | 3 (2-4) | 3.72 (0.63) | 4 (2-4) | <0.001 | 0.49 |
| 12. It is inappropriate to perform PPE on persons that will be my future colleagues | 3.38 (0.97) | 4 (0-4) | 3.63 (0.68) | 4 (0-4) | 0.018 | 0.23 |
| 13. To perform PPE is an appropriate practice for the education of a medical doctor (osteopath) | 3.80 (0.48) | 4 (2-4) | 3.85 (0.51) | 4 (0-4) | 0.424 |  |
| 14. To undergo PPE is an appropriate practice for the education of a medical doctor (osteopath) | 3.80 (0.48) | 4 (2-4) | 3.86 (0.34) | 4 (3-4) | 0.629 |  |
| 15. In performing PPE I (will) get useful feedback from my colleagues about my skill | 3.72 (0.53) | 4 (2-4) | 3.60 (0.61) | 4 (1-4) | 0.230 |  |
| 16. It is a sign of professionalism as a student to accept to perform and undergo PPE | 3.65 (0.66) | 4 (0-4) | 3.70 (0.55) | 4 (2-4) | 1.000 |  |
