# Supplementary material for: Perception of peer physical examination in two Australian osteopathy programs
Source: Chiropr Man Therap. 2016 Jul 11;24:21. doi: 10.1186/s12998-016-0102-2 (PMC4939704; doi:10.1186/s12998-016-0102-2)
Supplement: Additional file 2 — Ordinal Logistic Regression for Peer Physical Examination Questionnaire (PPEQ) items & demographics (DOCX 24 kb) [file 12998_2016_102_MOESM2_ESM.docx]

**Ordinal Logistic Regression for PPEQ items & demographics**

| **PPEQ item** | **T1** | **T2** |
| --- | --- | --- |
| 1. In general, I (will) feel comfortable when performing PPE on a colleague of mine | NS | NS |
| 2. In general, I (will) feel comfortable when a colleague performs PPE on me | NS | Age 20-25 years (OR 2.85, 95%CI [1.24 – 11.13])  Age 26 or over (OR 1.08, 95%CI [0.30 – 7.09]) |
| 3. I (will) feel embarrassed if I am undressed for PPE in front of my group of colleagues | Gender female (OR 2.91, 95%CI [1.44-6.29])  Previous (OR 2.07, 95%CI [1.19-6.48])  Australia (OR 2.80, 95%CI [1.33-12.93]) | Gender female (OR 1.91, 95%CI [1.44-6.29])  Age 20-25 years (OR 3.22, 95%CI [1.34-8.84])  Age 26 years of over (OR 1.36, 95%CI [0.23-2.77])  Australia (OR 13.19, 95%CI [2.61-68.71]) |
| 4. I (will) feel embarrassed if I am undressed for PPE in front of my teacher or tutor | Gender female (OR 2.88, 95%CI [1.60-6.48]) | Gender female (OR 1.84, 95%CI [0.91-4.05])  Age 20-25 years (OR 4.01, 95%CI [1.95-13.59])  Age 26 years of over (OR 1.24, 95%CI [0.52-5.98])  Australia (OR 9.20, 95%CI [2.15-48.81]) |
| 5. I am concerned of being a possible object of sexual interest during PPE | Age 20-25 years (OR 1.39, 95%CI [0.25 – 1.91])  Age 26 or over (OR 21.11, 95%CI [1.68 - 242.25])  Gender (OR 2.66, 95%CI [1.13 – 6.23])  Australia (OR 7.24, 95%CI [1.19 – 43.81])  Religion (OR 4.95, 95%CI [1.56 – 15.48]) | Age 20-25 years (OR 2.71, 95%CI [1.17 – 7.17])  Age 26 or over (OR 3.63, 95%CI [2.61 – 41.67])  Previous (OR 3.63, 95%CI [1.47 – 21.32])  Australia (OR 5.64, 95%CI [2.05 – 56.26]) |
| 6. I am concerned of experiencing possible sexual interest for my colleagues during PPE | Age 20-25 years (OR 1.95, 95%CI [1.40 – 5.36])  Age 26 or over (OR 4.48, 95%CI [1.11 – 22.42])  Australia (OR 4.52, 95%CI [1.23 – 25.27])  Religion (OR 3.28, 95%CI [1.19 – 9.48]) | NS |
| 7. I am concerned of experiencing possible sexual interest for my teacher or tutor during PPE | Age 20-25 years (OR 2.53, 95%CI [1.10 – 9.67])  Age 26 or over (OR 8.58, 95%CI [2.07 – 66.68])  Australia (OR 7.24, 95%CI [1.17 – 44.25]) | Age 20-25 years (OR 7.40, 95%CI [1.29 – 26.84])  Age 26 or over (OR 1635, 95%CI [large])  Australia (OR 9.11, 95%CI [1.19 – 157.59]) |
| 8. I (will) feel comfortable when performing PPE on a colleague of my same sex | NS | Age 20-25 years (OR 4.30, 95%CI [1.33 – 18.17])  Age 26 or over (OR 1.30, 95%CI [0.24 – 11.02]) |
| 9. I (will) feel comfortable when performing PPE on a colleague of the opposite sex than mine | NS | Age 20-25 years (OR 1.64, 95%CI [1.41 – 5.81])  Age 26 or over (OR 6.23, 95%CI [1.87 – 47.46]) |
| 10. I (will) feel comfortable when PPE is performed on me by a colleague of my same sex | NS | Age 20-25 years (OR 3.66, 95%CI [1.63 – 21.11])  Age 26 or over (OR 1.06, 95%CI [1.19 – 3.85]) |
| 11. I (will) feel comfortable when PPE is performed on me by a colleague of the opposite sex than mine | NS | NS |
| 12. It is inappropriate to perform PPE on persons that will be my future colleagues | Age 20-25 years (OR 4.17, 95%CI [1.75 – 14.73])  Age 26 or over (OR 3.09, 95%CI [1.19 – 16.28])  Religion (OR 3.18, 95%CI [1.01 – 9.48]) | Age 20-25 years (OR 2.97, 95%CI [1.75 – 14.73])  Age 26 or over (OR 1.06 , 95%CI [1.19 – 16.28]) |
| 13. To perform PPE is an appropriate practice for the education of a medical doctor (osteopath) | NS | NS |
| 14. To undergo PPE is an appropriate practice for the education of a medical doctor (osteopath) | NS | NS |
| 15. In performing PPE I (will) get useful feedback from my colleagues about my skill | Gender female (OR 2.07, 95%CI [1.22 – 5.36]) | Gender female (OR 1.93, 95%CI [1.10 – 4.80]) |
| 16. It is a sign of professionalism as a student to accept to perform and undergo PPE | NS | NS |
